# Editorial: Pathogen Genomics: Empowering Infectious Disease Surveillance and Outbreak Investigations

**DOI:** 10.3389/fpubh.2020.00179

**Published:** 2020-05-19

**Authors:** Marc J. Struelens, Vitali Sintchenko

**Affiliations:** ^1^European Centre for Disease Prevention and Control (ECDC), Solna, Sweden; ^2^Sydney Medical School and Marie Bashir Institute for Infectious Diseases and Biosecurity, University of Sydney, Sydney, NSW, Australia

**Keywords:** microbial genomics, whole genome sequencing, epidemiology, antimicrobial resistance (AMR), public health laboratory surveillance

Comparative microbial genomics analysis by high-throughput whole-genome sequencing (WGS) offers exquisite resolution for epidemiological investigations of infectious disease. This approach has revolutionized outbreak detection and monitoring of transmission dynamics of infectious agents and antimicrobial resistance across humans, animals, and environment. The objective of this Research Topic was to assemble articles on genomic epidemiological approaches to identify sources and mechanisms of transmission of infection and antimicrobial resistance. Leading experts discuss advances in fine-tuning the WGS laboratory workflows and bioinformatics for analyzing viral, bacterial, and protozoan genomic data as well as best available WGS data sharing and vizualization tools for infectious disease surveillance and control. The Research Topic consists of 13 articles on public health applications of comparative genomics of key human and zoonotic pathogens, including Original research, Reviews, Systematic Review and Curriculum, instruction, and pedagogy reports.

The ongoing COVID-19 pandemic underlines the crucial need for open access to advanced viral infectious disease surveillance systems that integrate genomic and epidemiological data on epidemic pathogens in real-time. Theys et al. review cutting-edge phylodynamic analysis tools and visualization solutions for translating these data into information for disease control decisions by public health and health policy professionals. They discuss bioinformatics platform use for temporal and spatial visualization through examples for tracking viral disease dissemination across populations and monitoring viral evolution and adaptation.

Hendriksen et al. describe the value of bacterial genomics for monitoring the global threat of antimicrobial resistance by efficient and rapid identification of genetic determinants of drug resistance. They review the operational characteristics, functionalities, strengths, and limitations of bioinformatics tools and knowledge bases accessible online for these purposes. They offer their expert perspective for standardization of analytical pipelines and database validation for more robust genomic-enhanced antimicrobial resistance surveillance.

Ribeiro Duarte et al. report on their e-learning and crowdsourcing pedagogic experience to explore expectations and opinions as well as educate academic, public health, and food safety professionals on the potential of microbial metagenomics in surveillance of pathogens and antimicrobial resistance. They ran a blended training exercise followed by massive open online interactive course to disseminate metagenomics knowledge and skills to a global audience of research and surveillance experts and gather their opinions. A majority of participants expected a slow transition to metagenomics for surveillance and food safety risk assessment subject to further harmonization of experimental protocols and interpretation of results.

The value of next-generation sequencing technologies for surveillance and study was reviewed for several human pathogens*. Clostridioides (Clostridium) difficile* is an important enteric pathogen in the healthcare setting where it causes both sporadic and epidemic infections with substantial morbi-mortality. It is increasingly frequent as a community-acquired pathogen although infection sources remain elusive. In a mini-review, Janezic and Rupnik summarize the WGS-based typing schemes for *C. difficile* and compare the merits of single nucleodide variant typing and core genome multilocus sequence typing (cgMLST) methods for surveillance and cluster investigations. They highlight how WGS-based studies help elucidating the global population structure of *C. difficile*, mapping the intercontinental spread of epidemic lineages, resolving relapses, and reinfections in recurrent disease and evaluating the effect of disease prevention measures. Knight and Riley review the diverse ecological reservoirs of *C. difficile* from a One-Health perspective. Micro-evolutionary studies are revealing how the open pan-genome of several successful zoonotic lineages that have adapted to different ecological niches. This foster their global spread between food animals and farm environment to humans under the selective pressure of antimicrobial use in livestock and human medicine.

van der Werf and Ködmön report on a systematic review of three international tuberculosis outbreak investigations that were supported by WGS-based typing. WGS data analysis from different sequencing platforms used the SNV mapping approach with diverse bioinformatics tools. WGS analysis was helpful in supporting evidence from epidemiological data for delineating outbreak-related-cases and excluding unrelated ones. Further standardization of WGS methodology and data sharing procedures is desirable for tuberculosis control.

Morris et al. assess the state-of-the-art genome sequencing methods for *Cryptosporidium* species identification and genotyping in the public health setting. Technical hurdles relate to genomic DNA extraction, sequencing depth, and assembly. Biological complexity is challenging due to sexual recombination of the parasite and multiplicity of infection in humans and animals. While WGS is not yet feasible for routine genotyping, the increasing volume of *Cryptosporidium* genome data available from diverse hosts and geographical sources is helping design novel genotyping markers and better understand its population diversity and virulence variation.

Seth-Smith and Egli have examined current evidence on high-resolution typing of *Corynebacterium diphtheriae* using WGS for surveillance of this re-emerged pathogen in low incidence settings and describe international networks supporting this new approach to the control of diphtheria. The authors review the phylogeny of this diverse species and explain how the timing of disease can be inferred from WGS data. They argue that de-centralized sequencing strategies with redundancy in sequencing capacities, followed by data exchange, may be a valuable future option, especially as WGS becomes more available and portable.

Several contributions to this Research Topic have focused the attention on the added value of genomic surveillance for controlling foodborne diseases. Chattaway et al. describe the transformational impact of WGS on reference microbiology practice and public health laboratory surveillance for Salmonella. They document experience from Public Health England and review challenges of implementing WGS as a routine country-wide reference laboratory service. Their experience started in 2014 and led to the radical transformation of public health practice based on the integrated and cross-disciplinary analysis and decision-making. The authors explain how this transformation led to improved accuracy of results, reduced turn-around times of reports and better recognition and monitoring of smaller and geographically dispersed outbreaks of common Salmonella serovars and outbreaks of prolonged duration. They outline the PHE approaches to the bioinformatics pipelines, detection of antimicrobial resistance in foodborne bacteria and integrated analysis of data. The authors also remind us about the essentiality of inter-agency sharing and comparisons of microbiological, epidemiological, and food chain analyses for effective food safety and control.

These messages are reinforced and expanded by Gerner-Smidt et al. from the US Centers of Disease Control and Prevention who presented compelling evidence for the One Health approach to foodborne surveillance. They argue that such an approach takes public health surveillance to the next level as many foodborne outbreaks ultimately originate from animal or environmental sources. The authors illustrate the power of this approach in helping to successfully solve several persistent community outbreaks, including polyclonal listeriosis associated with contaminated ice cream, multidrug resistant Salmonella Heidelberg linked to contaminated chicken, an outbreak caused by six serotypes of Salmonella associated with consumption of an imported herbal supplement, and multidrug resistant Campylobacter linked to contact with puppies sold by a specific pet store chain. Such outbreaks are significantly more complex than typical point source outbreaks and might be difficult to solve using traditional epidemiological approaches. This paper presents examples of how WGS surveillance enables flexible outbreak case definitions and efficient epidemiological traceback.

An insightful report from the Austrian Agency for Health and Food Safety led by Pietzka et al. demonstrates the potential of genome sequencing in identifying sources of outbreaks of listeriosis. The authors utilized a core genome MLST scheme based on 1,701 target genes to type over six thousand isolates of *Listeria monocytogenes* from human and food associated sources. The typing results helped to identify a community outbreak in eastern Austria and trace back the source of the outbreak to one meat-processing company. The whole-genome sequence based typing yielded better accuracy and higher discriminatory power than pulsed-field gel electrophoresis as well as higher laboratory throughput at a lower cost. These findings are of particulars relevance to public health microbiologists as the growing proportion of elderly citizens drives up listeriosis notifications across many countries in the EU and Northern America.

Seth-Smith et al. aim to improve our understanding of whole genome sequencing protocols employed for laboratory surveillance. The authors evaluated three popular library preparation protocols based on enzymatic fragmentation which are fast and require minimal amounts of genomic DNA. They provided in-depth analysis of WGS results obtained from libraries prepared by Nextera XT (Illumina), Nextera Flex (Illumina), and QIAseq FX (Qiagen) protocols using a set of 12 reference strains representing pathogenic bacteria with different DNA guanine-cytosine (GC) content. The results suggest that Nextera Flex and Qiaseq FX are less sensitive than Nextera XT to variable GC content. Interestingly, more alleles were detected in the cgMLST analysis with these two best library preparation protocols, producing better discrimination of closely related genomes. Furthermore, these protocols achieved a more complete representation of accessory genes and ensured the detection of every antibiotic resistance gene from short read data with coverage of 50 or higher.

We hope both public health professionals and clinicians will find this issue useful for their practice. The editorial team thanks external reviewers for their constructive criticism and hopes that this issue of Frontiers in Public Health will assist healthcare professionals and scientists involved in translational research and genomics-informed public health laboratory surveillance and will be of interest to everyone who is passionate about international efforts to control communicable diseases.

## Author Contributions

MS and VS jointly drafted and approved the final manuscript.

## Conflict of Interest

The authors declare that the research was conducted in the absence of any commercial or financial relationships that could be construed as a potential conflict of interest.

